# Tunable Surface Area, Porosity, and Function in Conjugated Microporous Polymers

**DOI:** 10.1002/anie.201905488

**Published:** 2019-07-17

**Authors:** Jie Chen, Wei Yan, Esther J. Townsend, Jiangtao Feng, Long Pan, Veronica Del Angel Hernandez, Charl F. J. Faul

**Affiliations:** ^1^ Department of Environmental Science and Engineering Xi'an Jiaotong University Xi'an Shaanxi 710049 P. R. China; ^2^ School of Chemistry University of Bristol Bristol BS8 1TS UK; ^3^ College of Environment and Resources Fuzhou University Fuzhou Fujian 350116 P. R. China; ^4^ Bristol Centre for Functional Nanomaterials University of Bristol Bristol BS8 1TL UK

**Keywords:** conjugated microporous polymers, Hansen solubility parameters, metal-catalyzed cross-couplings, tunable properties

## Abstract

Simple inorganic salts are used to tune N‐containing conjugated microporous polymers (CMPs) synthesized by Buchwald–Hartwig (BH) cross‐coupling reactions. Poly(triphenylamine), PTPA, initially shows a broad distribution of micropores, mesopores, and macropores. However, the addition of inorganic salts affects all porous network properties significantly: the pore size distribution is narrowed to the microporous range only, mimicking COFs and MOFs; the BET surface area is radically improved from 58 m^2^ g^−1^ to 1152 m^2^ g^−1^; and variations of the anion and cation sizes are used to fine‐tune the surface area of PTPA, with the surface area showing a gradual decrease with an increase in the ionic radius of salts. The effect of the salt on the physical properties of the polymer is attributed to adjusting and optimizing the Hansen solubility parameters (HSPs) of solvents for the growing polymer, and named the Beijing–Xi'an Jiaotong (BXJ) method.

Recently developed conjugated microporous polymers (CMPs) have advantages over other microporous organic polymers (MOPs), especially over covalent organic frameworks (COFs) and metal–organic frameworks (MOFs), including high physical and chemical stability, ease of synthesis, high surface areas, and high microporosity.^1^ Furthermore, the unique extended π‐conjugated structures of CMPs make them suitable for applications in energy storage and electronic devices.^2^ A wide variety of coupling reactions have been used to develop novel CMPs, including Sonogashira–Hagihara,1b Suzuki,^3^ Friedel–Crafts,^4^ Yamamoto,^5^ and Fe^3+^‐catalyzed oxidative polymerization.^6^ Recently, Buchwald–Hartwig (BH) coupling has been utilized in CMP design and synthesis, where C−N bonds are created in the polymer network by the palladium‐catalyzed cross‐coupling of (aryl)amines with aryl halides.^2^ This coupling approach also provides a simple route to develop N‐rich redox‐active CMPs, that is, 3D analogues of the well‐known redox‐active polymer poly(aniline).^2^ However, one significant drawback of CMPs synthesized by BH coupling reactions is the low surface areas (ca. 50 m^2^ g^−1^) obtained when compared with those synthesized by other coupling reactions.^2^ Moreover, they possess unexpectedly low micropore volumes when compared with structurally related poly(aryleneethynylene)s (PAEs).1b Careful analysis showed that the major difference between BH coupling reactions and other employed methods is the addition of salts. Specifically, in other reactions salts such as NiCl_2_, AlCl_3_, CuI, FeCl_3_, ZnCl_2_, and K_2_CO_3_ are extensively used as catalysts or bases while no inorganic salts are used in BH coupling reactions. We speculated that the addition of salts may be an important factor in enhancing porosity in CMPs. In this study we set out to explore the influence of the presence of salts: either to act as a template, or potentially adjusting the solubility parameters of the reaction solvent. These effects could lead to simple salts controlling, contributing and enhancing porosity, and tuning micropore size distributions in BH‐prepared CMPs, leading to a new generation of CMPs and applications.

We therefore report herein exquisite control over porous network properties enabled by the addition of simple salt species in the preparation of poly(triphenylamine), PTPA, networks by BH coupling (Scheme 1; see the Supporting Information, Table S1 for the radii of salt ions). We name this approach the Bristol–Xi'an Jiaotong (BXJ) method. Furthermore, we provide detailed analysis of the effects that lead to this unprecedented control, proposing general design rules related to solubility parameters for the control of porosity in a wide range of important N‐rich materials. To the best of our knowledge, the surface areas reported herein for PTPAs, tuned by the addition of small inorganic salts (that is, BXJ‐PTPAs), specifically LiNO_3_, or NaF, outperform most CMPs and MOPs (Supporting Information, Table S2 and S3) in terms of properties and functionality.^7^

**Scheme 1:**
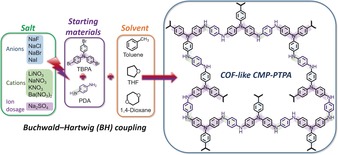
Synthetic route for the formation of salt‐tunable PTPA networks.

To investigate the scope of our BXJ approach and possibility to actively tune material properties (including porosity) of our model CMP, 0.5 mm of various sodium salts (that is, the same cation), including NaF, NaCl, NaBr, and NaI were used at first, with the results shown in Figure 1 a–c and Table 1 (see the Supporting Information, Table S3 for full details). The addition of salts during polymerization leads to macroscopic gelation of the reaction solution, which is also observed in CMP‐1 synthesis.1b Successful coupling reactions were verified by FTIR and solid‐state ^13^C cross‐polarization magic angle spinning nuclear magnetic resonance (SS ^13^C CP/MAS NMR; Supporting Information, Figure S1). The characteristic peaks assigned to the core and linker were detected in the FTIR spectra of all products (Supporting Information, Figure S4). Additionally, peaks at 710, 1004, and 1070 cm^−1^ (assigned to vibration frequencies originating from C−Br bonds), and the bands at 3400 and 3300 cm^−1^ (−NH_2_ stretching frequencies), are absent from the spectra of the products, demonstrating successful BH coupling.^2^, ^8^ SS ^13^C CP/MAS NMR spectra of the PTPA polymers show two main resonances at 141 and 127 ppm, which are assigned to the substituted phenyl carbons and unsubstituted phenyl carbons, respectively, fitting well with the simulated results (Supporting Information, Figure S5). Effective coupling was also confirmed by energy‐dispersive X‐ray spectroscopy (EDS) data (Supporting Information, Table S4), which shows the absence of Br in the products. Traces of salts were detected in the polymers using EDS.

**Figure 1:**
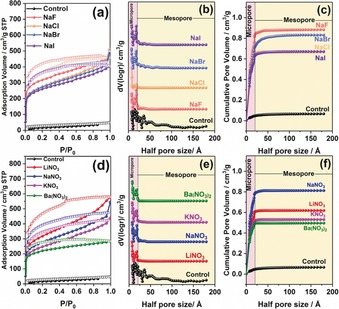
N_2_ adsorption and desorption isotherms, nonlocal density functional theory‐pore size distribution, and cumulative pore volume of the PTPA networks tuned by salts with different anions (a–c) and cations (d–f). The pink rectangular strips indicate the microporous region in the pore size distribution.

**Table 1:** Porosity parameters and CO_2_ uptake at 1 atm and 273 K of PTPA networks produced by the BXJ route with different anions and cations.

	Surface area^[a]^ [m^2^ g^−1]^	Total pore volume^[b]^ [cm^3^ g^−1^]	CO_2_ uptake^[c]^ [mmol g^−1^]	Ref.
control	58	0.066	0.70	this work
NaF	1134	0.89	3.23	this work
NaCl	1123	0.67	3.05	this work
NaBr	1114	0.83	3.44	this work
NaI	1075	0.69	3.60	this work
LiNO_3_	1152	0.62	2.78	this work
NaNO_3_	1028	0.82	3.48	this work
KNO_3_	873	0.53	2.43	this work
Ba(NO_3_)_2_	831	0.50	3.20	this work
CMP‐1	834	0.53	N.A.	1b
CTF‐1	791	0.40	N.A.	10
COF‐1	711	0.32	N.A.	11
MOF‐1	516	0.29	0.86	12

[a] Surface area calculated from the N_2_ adsorption isotherm using the Brunauer–Emmett–Teller method. [b] The total pore volume calculated from the desorption branch of the N_2_ isotherm using the NL‐DFT method. [c] CO_2_ uptake at 273 K, 1 atm.

N_2_ adsorption isotherms were used to determine the influence of salts on the porosity of the networks (Figure 1 and Table 1; see the Supporting Information, Table S3 for full details). Porosity data of typical COFs and MOFs are also provided for comparison. The PTPA networks without salt show Type II nitrogen adsorption isotherms.^9^ Such behavior indicates some outer surface area that most likely results from interparticular voids or interparticulate porosity, assigned to meso‐ and macropores, which are commonly found in CMPs synthesized by BH coupling reactions.^2^, ^8^ Remarkably, our BXJ‐PTPA polymer networks show typical Type I nitrogen adsorption isotherms, demonstrating that large numbers of micropores were produced by adding salt.1b

The BET surface area of PTPA notably improved almost 20 times from 58 m^2^ g^−1^ to 1134 m^2^ g^−1^ by the addition of NaF, with a gradual decrease to 1075 m^2^ g^−1^ with an increase of the ionic radii of the anions. This trend suggests a route to careful tuning of the surface area. Furthermore, the nonlocal density functional theory (NLDFT) pore size distribution (PSD) shows distinct differences before and after salt treatment. The PSD is noticeably modified from a broad distribution to pure micropores only (with half pore size of 0.7 nm). The total pore volume, the micropore volume and the ultramicropore volume are greatly increased from 0.066 cm^3^ g^−1^, 0.046 cm^3^ g^−1^, and 0.026 cm^3^ g^−1^ to 0.89 cm^3^ g^−1^, 0.83 cm^3^ g^−1^, and 0.41 cm^3^ g^−1^, respectively, by the addition of NaF, thus providing our amorphous BXJ‐PTPA materials attractive properties. The outcome of the BXJ approach is that the surface area is not only radically modified, but significantly enhanced; these materials now resemble COFs and MOFs very closely. Our BXJ approach provides a simple but very efficient route for the synthesis of a next generation of CMPs with properties very similar or, in some cases, better than the well‐known COFs and MOFs. However, unlike COFs, the XRD patterns (Supporting Information, Figure S6) show amorphous structures for all the networks.^11^ Only slight differences in the PSD shape are noted when using salts of different anions, suggesting that the actual salt employed has little influence on the PSD.

Next, to confirm the wider applicability of our BXJ method, nitrate salts (with different cations), including LiNO_3_, NaNO_3_, KNO_3_, and Ba(NO_3_)_2_ were used to tune the porosity of BXJ‐PTPAs, as shown in Figure 1 d–f and Table 1 (with further details in the Supporting Information, Table S3). Similar to the results of our initial investigations using salts with varying anion size, the BET surface area of PTPA increases to 1152 m^2^ g^−1^ with the addition of LiNO_3_, and then decreases with increasing cationic radius of the salt. The NLDFT‐PSD of the BXJ‐PTPA shows that it is microporous in nature, with total pore volume, micropore volume and ultrapore volume growth ascertained to be 0.82 cm^3^ g^−1^, 0.78 cm^3^ g^−1^, and 0.43 cm^3^ g^−1^ maximum, respectively. However, there is little difference in the shape of the PSD curves when different nitrate salts are used, indicating that the PSD is mainly determined by the strut lengths of the linker.1b, 13 XRD investigations again show amorphous networks, confirming that long‐range order is not a prerequisite for fine BXJ control over the microporous organic network properties.1b Unlike the surface area, there is no obvious trend for the pore volume when increasing the ionic radius of the applied salts, suggesting the pore volume could not be finely controlled by the salt even though the surface areas of polymers can be markedly improved by the salts. The formation and control of pores with different volumes appears to be more complex. Owing to the increase in pore volume, the CO_2_ uptake (that is, function) of PTAP at 1 atm and 273 K also dramatically improved by >500 % from 0.70 mmol g^−1^ to 3.60 mmol g^−1^ by the addition of NaI (Supporting Information, Table S3 and Figure S16, see also therein for further information).

To further understand the observation that salts could tune the porosity and surface area of the CMPs, FTIR, SS ^13^C NMR, XRD, SEM, and solid‐state UV/Vis NIR spectroscopy were used to investigate the products from our BXJ investigations. Interestingly, the FTIR spectra of the products before and after salt addition (Supporting Information, Figure S1 a,b) are almost identical, with the only difference being the presence of a small peak situated at around 1650 cm^−1^, which is ascribed to the salts (Supporting Information, Figure S8; hard to remove from the polymers due to the microporous nature, as shown in Figure S9 and detected by EDS, Table S4). Furthermore, identical SS ^13^C NMR and XRD spectra of the materials before and after BXJ treatment were obtained (Supporting Information, Figure S1 and Figure S6), suggesting that the porosity of the products is tuned by the presence of the chosen salts without any change to the actual chemical structure of the polymers.

SEM investigations into micro‐ and macroscopic features showed interesting changes before and after BXJ treatment (images shown in the Supporting Information, Figure S10): the uneven particulate‐like morphology of PTPA becomes smooth and almost foam‐like after the addition of salts.1b This morphology confirmed that the principle of our BJX method was not related to classical salt templating.^14^ As noted earlier, and in the Supporting Information, the addition of salts during polymerization leads to macroscopic gelation of the reaction solution. Therefore, it could follow that the use of salts influence the phase separation of PTPA from the reaction mixture, resulting in the difference in morphology and porosity (but with no change in chemical composition).^13^ UV/Vis NIR spectra presented in Figure 2 a,b show typical PANi (EB) peaks at around 650 nm in all samples, attributed to the π–π* transition of the quinoid group.^15^ However, there is an obvious and significant bathochromic shift for products tuned by the addition of salts in our BXJ approach (from 676 to 841 nm; see the Supporting Information, Table S5 and Figure S11), which is not only directly related to an increase in surface area, but also conjugation length (with no change in chemical structure, as already shown).^16^ Furthermore, the thermal stability of the PTPA is enhanced after salt addition (Supporting Information, Figure S2). Moreover, the X‐ray photoelectron spectroscopy (XPS) spectra of the PTPA networks before and after BXJ treatment (Supporting Information, Figures S12–S14) show the amount of imine increases regularly with the surface area of the polymer, supporting larger conjugation lengths and higher polymerization degrees of the polymers.^17^

**Figure 2:**
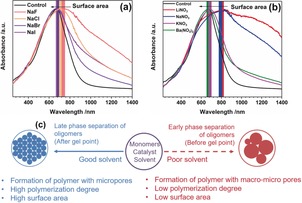
a),b) UV/Vis spectra of PTPA networks tuned by salts with different anions (a) and cations (b); c) the influence of compatibility between solvent and polymer on the porosity and surface area of the polymer network.

By summarizing the presented results, it appears that the manner by which salts tune the porosity of CMPs is similar to the way that porogens tune the physical structure of a wide variety of resins, including poly(urethane) and poly(divinylbenzene) (PVB) resins.^18^ As a general guide, solvents with poor thermodynamic compatibility and weak matching of their Hansen solubility parameters (HSPs) with the resultant polymer networks could result in the formation of microgels and early phase separation, leading to large average diameter pores and low BET surface areas.18a, 19 Solvents with good compatibilities with the polymer networks, in contrast, could contribute to phase separation at a much later stage during the polymerization, thus producing a polymer with lower average pore diameters and a larger surface area (Figure 2 c; see the Supporting Information for a detailed discussion and information about HSPs).18a Addition of salts acts similarly to the addition of porogens, helping to adjust and match the HSPs between the solvent and polymers, preparing CMPs with high surface areas and narrow PSDs.

To verify this hypothesis, we calculated HSPs for PTPA, with results shown in the Supporting Information, Figure S15 and Table S6 (see also the Supporting Information for detailed discussion). The total solubility parameter (*δ*_T_) of PTPA and solvents are shown in Table 2. It can be seen that the difference of *δ*_T_ between THF and PTPA (|*δ*_T_|, |*δ*_T_|=|*δ*_T, THF_ −*δ*_T, PTPA_|) before salt tuning was 3.1. According to the HSP theory, solvents show good compatibility with the polymer when the difference in solubility parameter (|*δ*_T_|) <1.^20^ THF was therefore not a suitable solvent before salt tuning. However, salts could increase the permanent dipole interactions (*δ*_P_) and the hydrogen‐bonding interactions (*δ*_H_) of the solvent owing to their influence on the ionic strength in the solvent, leading to the decrease of |*δ*_T_|. With this mechanism in mind, the influence of the ionic radii on the surface area could be well explained. The electronegativity, the *δ*_P_ and the *δ*_H_ of the ions reduce with the increase of ionic radius of the salt.^21^ It is therefore possible, through the addition of a salt, to tune the HSPs of the solvent, which, in turn, will match that of a good solvent for the growing polymer. Ions with smaller radii should have a greater influence on the *δ*_P_ and *δ*_H_ of the solvent by adjusting the polarity and hydrogen bonding of the solvents, thus resulting in a more effective adjustment of the compatibility of the HSPs with the polymer.

**Table 2:** Difference of total Hansen solubility parameter *δ_T_* between solvents and the PTPA.

	*δ*_T_	|*δ*_T_|=|*δ*_T, solvent_−*δ*_T, PTPA_|
toluene	18.2	4.4
THF	19.5	3.1
1,4‐dioxane	20.5	2.1
PTPA	22.6	–

Furthermore, if the suggested mechanism is applicable, the HSPs could be over‐tuned if too much salt is added and should then lead to a lowering of surface areas. The effect of ion dosage on the porosity and surface area of PTPA was therefore investigated (Supporting Information, Figure S7 and Table S3). The table shows the attempt to over‐tune the HSPs of the solvent by adding excess salt. The surface area of PTPA initially increases as the amount of Na_2_SO_4_ increases, and then gradually decreases when the dosage reaches 0.75 mm. These results fit with the HSPs of the solvent being over‐tuned, suggesting the BXJ approach of adding salts strongly supports the proposed mechanism.

To provide further support and show the general applicability of our approach, the influence of the HSPs of different solvents on the porosity and surface area of PTPA were also investigated (Table 2; Supporting Information, Figure S7, Table S3; see also therein for more details). It shows that the closer the HSPs of the solvent was to the calculated HSP of PTPA (|*δ*_T_|_(Dioxane‐PTPA)_< |*δ*_T_|_(THF‐PTPA)_< |*δ*_T_|_(Toluene‐PTPA)_), the higher the surface area was obtained for PTPA (*S*_BET, (Dioxane‐PTPA)_> *S*_BET, (THF‐PTPA)_> *S*_BET, (Toluene‐PTPA)_). These results showed that the surface area of PTPA could also be tuned through tuning the HSPs of the reaction solvent, which further supports the proposed mechanism in this study.^22^

In summary, full synthetic control of the surface area and porosity of PTPA CMPs by the addition of salts in our BXJ approach is reported in this communication. For the first time, we determine that the use of salts, and thus tuning of HSPs, is an important factor to acquire CMPs with high surface areas and well‐defined micropores; the BET surface area of a model CMP, PTPA, could be radically improved from 58 m^2^ g^−1^ to a maximum of 1152 m^2^ g^−1^. Importantly, control is exerted over surface areas by adjusting the ionic radii of the salts. The broad PSD of PTPA could additionally be tuned to a narrow micropore range distribution, similar to that of COFs and MOFs. This discovery shows that our BXJ approach provides a facile avenue to full control over CMPs: it opens a new low‐cost and simple route for the control and optimization of the properties (surface area and porosity) and function of a broad class of N‐rich CMPs, which is bound to impact on energy storage materials and CO_2_ capture and conversion, and thus to contribute to scientific efforts to address these global challenges.

## Conflict of interest

The authors declare no conflict of interest.

## Supporting information

As a service to our authors and readers, this journal provides supporting information supplied by the authors. Such materials are peer reviewed and may be re‐organized for online delivery, but are not copy‐edited or typeset. Technical support issues arising from supporting information (other than missing files) should be addressed to the authors.

Supplementary
