# Supplementary material for: Tunable Surface Area, Porosity, and Function in Conjugated Microporous Polymers
Source: Angew Chem Int Ed Engl. 2019 Jul 17;58(34):11715–9. doi: 10.1002/anie.201905488 (PMC6771584; doi:10.1002/anie.201905488)
Supplement: Supplementary file 1 — Supplementary [file ANIE-58-11715-s001.pdf]

Angewandte Chemie
Eine Zeitschrift der Gesellschaft Deutscher Chemiker

Supporting Information

Tunable Surface Area, Porosity, and Function in Conjugated Microporous Polymers

*Jie Chen, Wei Yan, Esther J. Townsend, Jiangtao Feng, Long Pan, Veronica Del Angel Hernandez, and Charl F. J. Faul**

anie_201905488_sm_miscellaneous_information.pdf

CONTENTS

Section S1. Experimental Section

S1.1 Chemicals

S1.2 Synthesis of polytriphenylamine (PTPA) CMPs without salts (control)

S1.3 Synthesis of polytriphenylamine (PTPA) CMPs with salts of different anions or cations

S1.4 Synthesis of polytriphenylamine (PTPA) CMPs with different amounts of Na_2SO_4

S1.5 Synthesis of polytriphenylamine (PTPA) CMPs in different solvents

S1.6 Characterization

Section S2. Supplementary information

S2.1 Introduction of Hansen solubility parameters

S2.2 Calculation of Hansen solubility parameters of polymers

Section S3. Results and discussion

S3.1 Physical properties of the PTPAs before and after salt adding

S3.2 Verification of higher polymerization degree of the PTPAs using XPS

S3.3 Discussion on HSPs of the PTPA in various conditions

S3.4 Comparison of PTPA tuned by salts with CMP-1

Section S4. Supplementary materials

S4.1 Supplementary figures

S4.2 Supplementary tables

S1. Experimental Section

S1.1 Chemicals

(Linker) phenylenediamine; (Core) tris(4-bromophenyl) amine; (Catalyst) bis(dibenzylideneacetone) palladium(0) ($Pd(dba)_2$, 63.9-78.1% carbon, 16.6-20.4% palladium); (Ligand) 2-dicyclohexylphosphino-2’,4’,6’-triisopropylbiphenyl (XPhos, 97%); (Base) sodium tert-butoxide (NaOtBu, 97%); (Salt) NaF, NaCl, NaBr, NaI, $LiNO_3$, $NaNO_3$, KNO_3, $Ba(NO_3)_2$ and Na_2SO_4 were of AR grades, which were purchased from Sigma-Aldrich, UK and were used as received.

S1.2 Synthesis of polytriphenylamine (PTPA) CMPs without salts (control)

A Schlenk tube was charged with a tris(4-bromophenyl) amine (0.5 mmol), phenylenediamine (0.33 mmol to obtain the 1.5:1 ratio of core to linker), $Pd(dba)_2$ (0.03 mmol, 4 mol%), XPhos (0.045 mmol) and NaOtBu (3.5 mmol) and was placed under a nitrogen atmosphere.[1] Anhydrous THF (30 mL) was added and the reaction mixture was heated under stirring at 65 ℃. After 48 hours, the mixture was cooled to room temperature. The products were then washed with 200 mL $CHCl_3$, ethanol, methanol and boiling water, to remove the catalyst, impurities and oligomers, followed by a 72 h Soxhlet extraction with methanol for 24 h, THF for 24 h and chloroform for 24 h, respectively.

S1.3 Synthesis of polytriphenylamine (PTPA) CMPs with salts of different anions or cations

A Schlenk tube was charged with a tris(4-bromophenyl) amine (0.5 mmol), phenylenediamine (0.33 mmol to obtain the 1.5:1 ratio of core), $Pd(dba)_2$ (0.03 mmol, 4 mol%), XPhos (0.045 mmol), NaOtBu (3.5 mmol) and salts (0.5 mmol NaF, NaCl, NaBr, NaI, $LiNO_3$, $NaNO_3$, KNO_3 or $Ba(NO_3)_2$) and was placed under a nitrogen atmosphere. Anhydrous THF (30 mL) was added and the reaction mixture was heated under stirring at 65 ℃. After 48 hours, the mixture was cooled to room temperature. The products were then washed with 200 mL $CHCl_3$, ethanol, methanol and boiling water to remove the catalyst, salt and oligomers, followed by a 72 h Soxhlet extraction with methanol for 24 h, THF for 24 h and chloroform for 24 h, respectively.

S1.4 Synthesis of polytriphenylamine (PTPA) CMPs with different amounts of Na_2SO_4

A Schlenk tube was charged with a tris(4-bromophenyl) amine (0.5 mmol), phenylenediamine (0.33 mmol to obtain the 1.5:1 ratio of core to linker), $Pd(dba)_2$ (0.03 mmol, 4 mol%), XPhos (0.045 mmol), NaOtBu (3.5 mmol) and Na_2SO_4 (0.33, 0.5, 0.75, 1.00, 1.50 or 2.00 mmol) and was placed under a nitrogen atmosphere. Anhydrous THF (30 mL) was added and the reaction mixture was heated under stirring at 65 ℃. After 48 hours, the mixture was cooled to room temperature. The products were then washed with 200 mL $CHCl_3$, ethanol, methanol and boiling water to remove the catalyst, salt and oligomers, followed by a 72 h Soxhlet extraction with methanol for 24 h, THF for 24 h and chloroform for 24 h, respectively.

S1.5 Synthesis of polytriphenylamine (PTPA) CMPs in different solvents

A Schlenk tube was charged with a tris(4-bromophenyl) amine (0.5 mmol), phenylenediamine (0.33 mmol to obtain the 1.5:1 ratio of core to linker), $Pd(dba)_2$ (0.03 mmol, 4 mol%), XPhos (0.045 mmol), NaOtBu (3.5 mmol) and Na_2SO_4 (0.5 mmol) and was placed under a nitrogen atmosphere. Anhydrous solvent (30 mL, Toluene, THF or Dioxane) was added and the reaction mixture was heated under stirring at 65 ℃. After 48 hours, the mixture was cooled to room temperature. The products were then washed with 200 mL $CHCl_3$, ethanol, methanol and boiling water to remove the catalyst, salt and oligomers, followed by a 72 h Soxhlet extraction with methanol for 24 h, THF for 24 h and chloroform for 24 h, respectively. For comparison, the samples synthesized in different solvents without salts were obtained through the same way as above but without adding 0.5 mmol of Na_2SO_4.

S1.6 Characterization

A PerkinElmer Spectrum 100 spectrometer was used to obtain the attenuated total reflection Fourier transform infrared (ATR FT-IR) spectra. Thermal gravimetric analysis (TGA) was performed on a TGA Q500 apparatus under a nitrogen atmosphere with a heating rate of 10 ℃/min. The ^{13}C CP/MAS NMR spectra were performed on a Varian VNMRS-600 spectrometer using a spin rate of 6800 Hz. N_2 adsorption/desorption isotherms and CO_2 adsorption studies were performed on a Quantachrome Quadrasorb instrument after degassing the samples under high vacuum at 150 ℃ for 5 h. The Brunauer-Emmett-Teller (BET) model

were used to calculate the specific surface areas using the adsorption branches of the N_2 isotherms in the relative pressure range from 0.05 to 0.20 at 77 K. The pore size distribution was calculated from the desorption branch of the N_2 isotherms using the nonlocal density functional theory (NL-DFT). Scanning electron microscope (SEM) images and energy dispersive X-ray spectroscopy (EDS) results were acquired on a JEOL 5600LV SEM microscope. Solid-state ultraviolet-visible near-infrared (UV−vis/NIR) spectra were obtained using a Shimadzu UV-2600 spectrometer. Powder X-ray diffraction (XRD) patterns were obtained with a Bruker D8 Advance diffractometer using Cu Kα radiation (2θ = 5 °-80 °, 40 kV, 30 Ma). X-ray photoelectron spectroscopy (XPS) spectra were recorded on a Kratos Axis Ultra DLD with an Almonochromatic X-ray source (1486.71 eV). Transmission electron microscopy images (TEM) were recorded on a JEM model 2100 electron microscope.

S2. Supplementary information

S2.1 Introduction of Hansen solubility parameters [2]

The solubility parameter approach proposed by Hansen for predicting polymer solubility and the compatibility of solvent for polymer synthesis has been widely used. There are three main types of interactions in common organic materials. The first is the nonpolar interactions (E_D). It is derived from atomic forces and has also been called dispersion interactions. Therefore, it is related to the molar volumes of the solvent. This parameter could not be tuned by salts. The second type of cohesion energy, namely the polar cohesive energy (E_P), is caused by permanent dipole–permanent dipole interactions. $E_P = 37.4\ (DM)*V^{1/2}$, where DM is the dipole moment, V is the molar volume. The third major cohesive energy source is hydrogen bonding, E_H, which is related to the polar and dispersion energies of vaporization from the total energy of vaporization. In this respect, hydrogen bonding resembles the polar interactions. Therefore, E_P and E_H could be tuned and should increase with the electronegativity of the salts. Generally speaking, these two solubility parameters could be tuned by various salts and solvents due to the change of dipole–permanent dipole interactions and hydrogen bonding.

The equation governing the Hansen parameters is that the total cohesion energy, E, which should be the sum of the individual energies.

$E = E_D + E_P + E_H$

the total solubility parameter could be acquired by dividing the molar volume as follows,

$E/V = E_D/V + E_P/V + E_H/V$

or

$\delta_T^2 = \delta_D^2 + \delta_P^2 + \delta_H^2$

To evaluate the compatibility of Hansen solubility parameters between solvent and polymer, the difference between δ_T two materials of $|\delta_T|$ ($|\delta_T| = |\delta_{T1} - \delta_{T2}|$) was applied.

If $|\delta_T| < 1$, then the solvent could be a good solvent; if $1 < |\delta_T| < 3$, then the solvent could be an intermediary solvent; otherwise, the solvent is a poor solvent and not suitable for synthesis.

S2.2 Calculation of Hansen solubility parameters of polymers

The Hansen solubility parameter of PTPA was experimentally determined by the dissolution method according to the literature.[3] The Hansen solubility parameters of PTPA were experimentally obtained as solubility parameters associated with the solvent that exhibited the highest dispersion concentration. Specifically, PTPA before salt tuning was dispersed into 13 solutions of different and known solubility parameters respectively (including hexane, ethyl acetate, diethyl ether, ethanol, toluene, dichloroethane, chloroform, acetone, isopropanol, THF, acetonitrile, water and 1,4-dioxane) by a high-pressure jet mill followed by ultra-sonication. The acquired dispersions were stabilized for over 24 hours, and the concentration of the PTPA in the supernatant of the dispersions was then determined by the Beer-Lambert-Bouguer law based on measured absorbance at 676 nm, which was the maximum absorbance of PTPA. Through this process, the equilibrium concentrations of the PTPA in each of the 13 solutions with different δ_T could be obtained. The concentration was plotted versus the solubility parameters. The solubility parameter peak positions were estimated with the statistical method B-Spline fitted by OriginPro 2017 software, according to the literature report. [3] Thus, Hansen solubility parameters of the PTPA could be well estimated. Through this method, the Hansen solubility parameter of PTPA was experimentally determined in **Figure S15**.

S3 Results and discussion

S3.1 Physical properties of the PTPAs before and after salt adding

PTPA networks were synthesized through BH coupling reactions, starting from tris(4-bromophenyl)amine and phenylenediamine (**Scheme 1**). A broad range of salts of different anionic or cationic radii, namely NaF, NaCl, NaBr, NaI or $LiNO_3$, $NaNO_3$, KNO_3, $Ba(NO_3)_2$ (at 0.5 mM concentration) were then added to the reaction mixtures and explored for their ability to tune the physical properties of the PTPA networks (see **Table S1** for radii). The polymer is initially a light brown colour when under an inert N_2 atmosphere during the polymerization reaction, but gradually turns dark blue with increased exposure to air, in a similar manner to pure poly(aniline).[4] The obtained polymers are insoluble in common organic solvents including toluene, THF, dioxane, dichloromethane, chloroform, ethanol and methanol, exhibiting their robustness and high degree of cross-linking.[1] The products also exhibit significantly higher thermal stabilities after addition of salt (T_{dec}>250 ℃ before the addition of salt vs. T_{dec}>500 ℃ after addition of salt, Please see **Figure S2**). Interestingly, some of the networks we resynthesized, like CMP-1,[5] show macroscopic gelation of the solution after salt addition during the polymerization reaction in the THF. The gels are retained upon washing with chloroform, but fragment into powders upon washing with methanol. The powders will swell when treated with solvents such as chloroform (**Figure S3** for full details). This phenomenon does not occur when the PTPA is polymerized without the addition of salts; instead, a powder-like precipitate is obtained, which does not swell upon washing with any solvents. In addition, product yields obtained after the **BXJ** process reached 99% (after a 72 h Soxhlet extraction with methanol for 24 h, THF for 24 h and chloroform for 24 h, respectively). The maximum yields obtained for polymers prepared without salt tuning, however, is only 52%.

S3.2 Verification of higher polymerization degree of the PTPAs using XPS

The typical polyaniline XPS spectra shape were observed in the spectra of the PTPA networks, suggesting the polyaniline structure in the PTPA networks. In addition, the ratio of the imine increase with the surface area (please see the ratio of imine to amine in the N1s spectra and the ratio of peak area at around 286.4 eV which is assigned to the imine carbon), indicating higher

polymerization degree of the polymers. [6]

S3.3 Discussion on HSPs of the PTPA in various conditions

In general, the polymerization process of PVB resins involves simultaneous vinyl polymerization, cross-linking, phase separation, microgel fusion, aggregation and pore in-filling, which results in a broad PSD ranging from micropores to macropores (as found for our CMPs). [7] Many studies have used organic solvents as porogens to tune the phase separation of PVB resins during polymerization, achieving control over the pore diameter and corresponding internal surface area.[7-8] As a general guide, solvents with poor thermodynamic compatibility and low matching of their Hansen solubility parameters (HSPs) with the resultant polymer networks could result in the formation of microgels and early phase separation, leading to large average diameter pores and low BET surface areas.[7, 9]

The Hansen solubility parameters of PTPA were experimentally estimated in **Figure S15** using the method provided in **Section S2.2** and literature. [3] From **Table S6** and **Table 2**, it can be seen that the difference of the Hansen solubility parameter ($|\delta_T|$) between the solvents applied in this study and PTPA was larger than 1, suggesting that the solvents were not good enough for PTPA synthesis. Therefore, low surface area and broad PSD of PTPA synthesized in these solvents could be observed before salt addition (**Figure S7** and **Table S3**). However, the surface area of the PTPA increases with the decrease of the difference of the Hansen solubility parameter ($|\delta_T|$) between the solvents applied in this study (**Table 2** and **Table S3**, $S_{BET,\ \text{(Dioxane-PTPA)}} > S_{BET,\ \text{(THF-PTPA)}} > S_{BET,\ \text{(Toluene-PTPA)}}$; $|\delta_T|_{\text{(Dioxane-PTPA)}} < |\delta_T|_{\text{(THF-PTPA)}} < |\delta_T|_{\text{(Toluene-PTPA)}}$)) can be still observed, indicating that mechanism of Hansen solubility parameter proposed in this study is well applicable. After the salt adding, the permanent dipole interactions (δ_P) and the hydrogen-bonding interactions (δ_H) of the solvent were increased by salts due to their influence on the ion strength of the solvent, leading to the decrease of the $|\delta_T|$ between polymer and solvent to be less than 1 and leading the solvent to be a good solvent for PTPA. As such, solvents with good thermodynamic compatibility and higher matching of their Hansen solubility parameters (HSPs) with the resultant polymer networks could result in the late phase separation, leading to uniform micropores and high BET surface areas.[7, 9]

S3.4 Comparison of PTPA tuned by salts with CMP-1

CMP-1, previously synthesized by Jiang and Cooper [5, 10] using a Sonogashira-Hagihara coupling, has a similar chemical structure to the polymers prepared in this investigation, and displayed similar properties such as high surface area (834 m^2/g), well defined PSD (micropores only) and an amorphous nature. However, the authors ascribed these results to the novel rigid structure of poly(aryleneethynylene) (PAE), and did not consider the effect of the addition of salt (CuI in this case) to the starting materials on the porosity of the PAE polymer. It could be assumed that the PAE networks synthesized by Jiang could have low surface areas and broad PSDs, similar to **PTPA**, if CuI or other salts were not used.

S4 Supplementary materials

S4.1 Supplementary figures

S4.1.1 FTIR and SS ^{13}C CP/MAS NMR of the PTPA tuned by salts

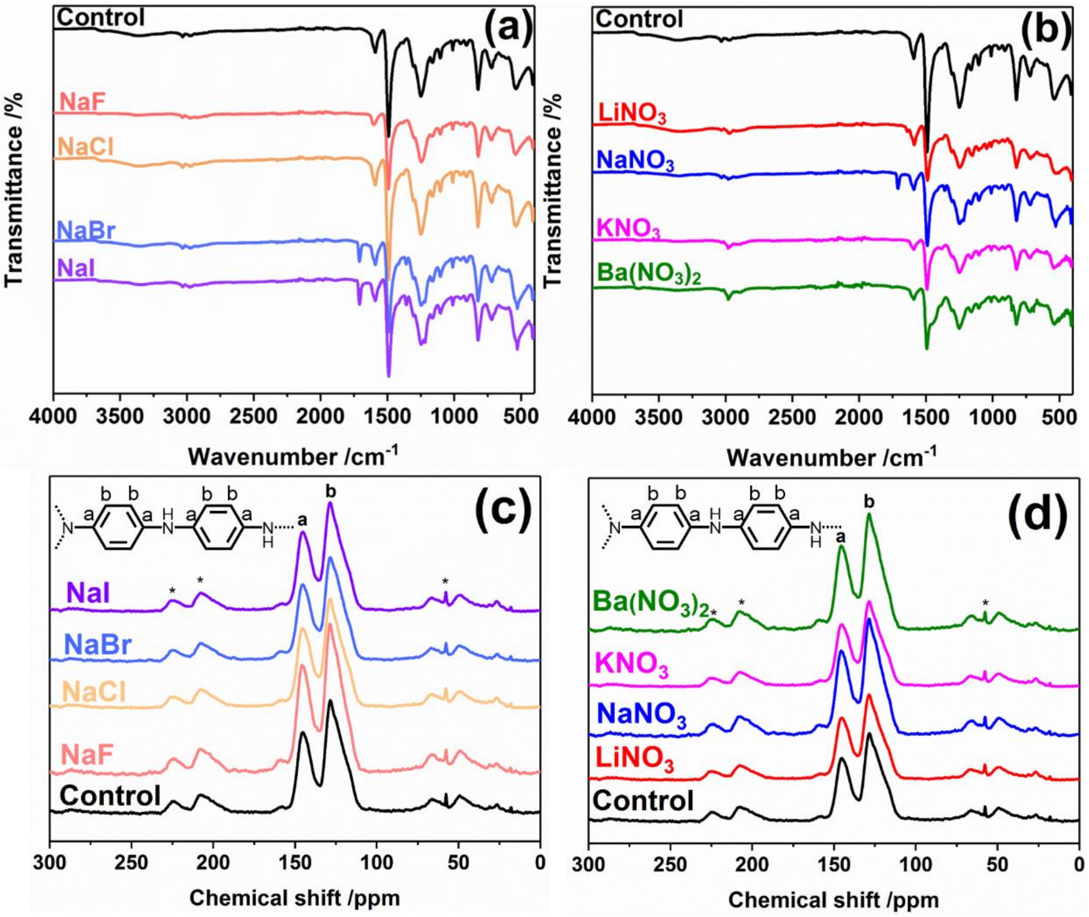

Figure S1 FTIR spectra of the PTPA networks produced applying the BXJ approach, with different anions (a) and cations (b); SS ^{13}C CP/MAS NMR spectra of the PTPA networks tuned by salts with different anions (c) and cations (d) (asterisks mark spinning side bands).

S4.1.2 Thermal stability of the PTPA tuned by salts

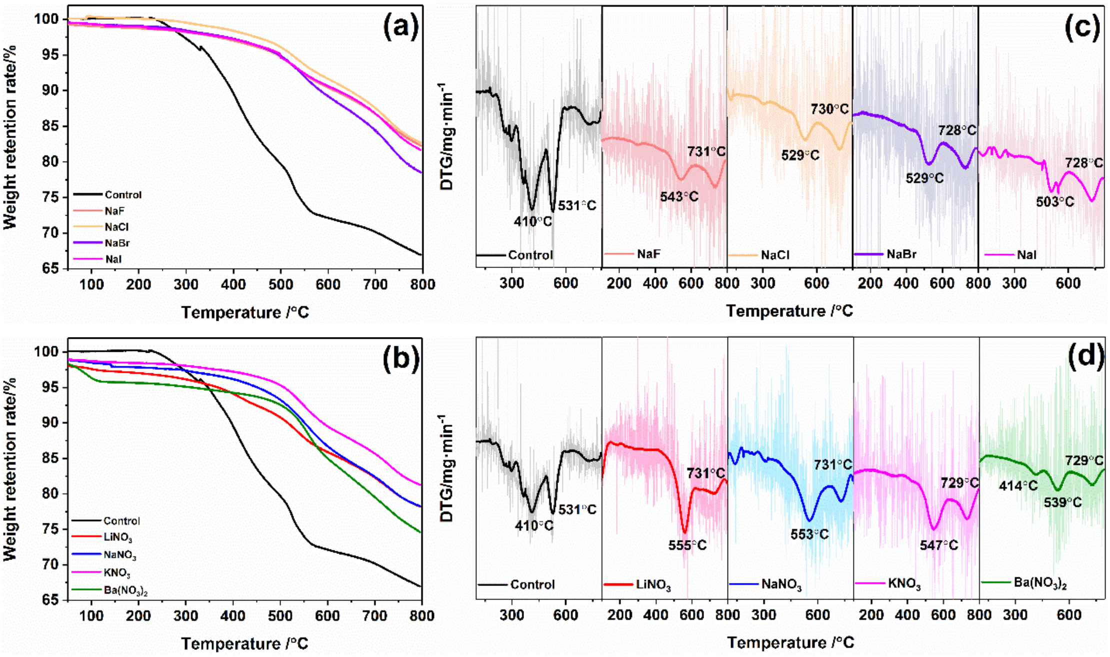

Figure S2 TGA curve (a,b) and DTG curve (c,d) of the PTPA before and after salt tuning.

S4.1.3 Swelling property of the PTPA tuned by salts

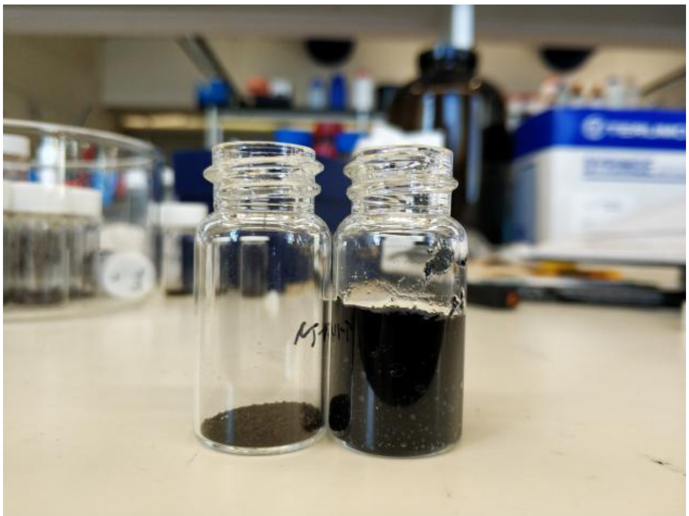

Figure S3 Photos of 200 mg of pure PTPA (left) and NaF-tuned PTPA (right) after adsorption and removal of chloroform using filtration.

S4.1.4 Characterization of the structure of the PTPA tuned by salts

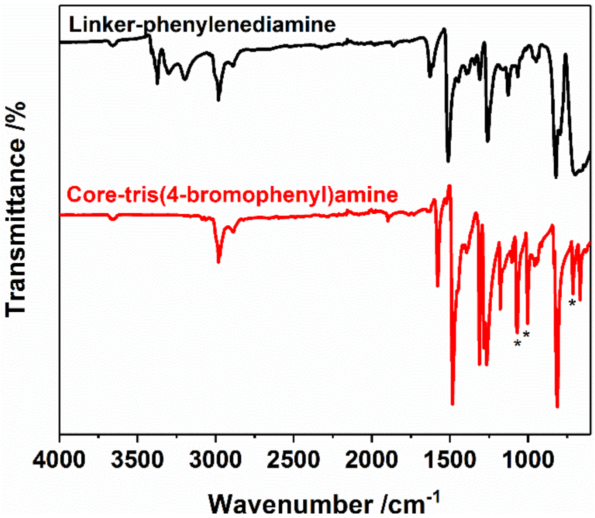

Figure S4 FTIR spectra of core (tris (4-bromophenyl)amine) and linker (phenylenediamine) (asterisks mark the bands assigned to C-Br bond).

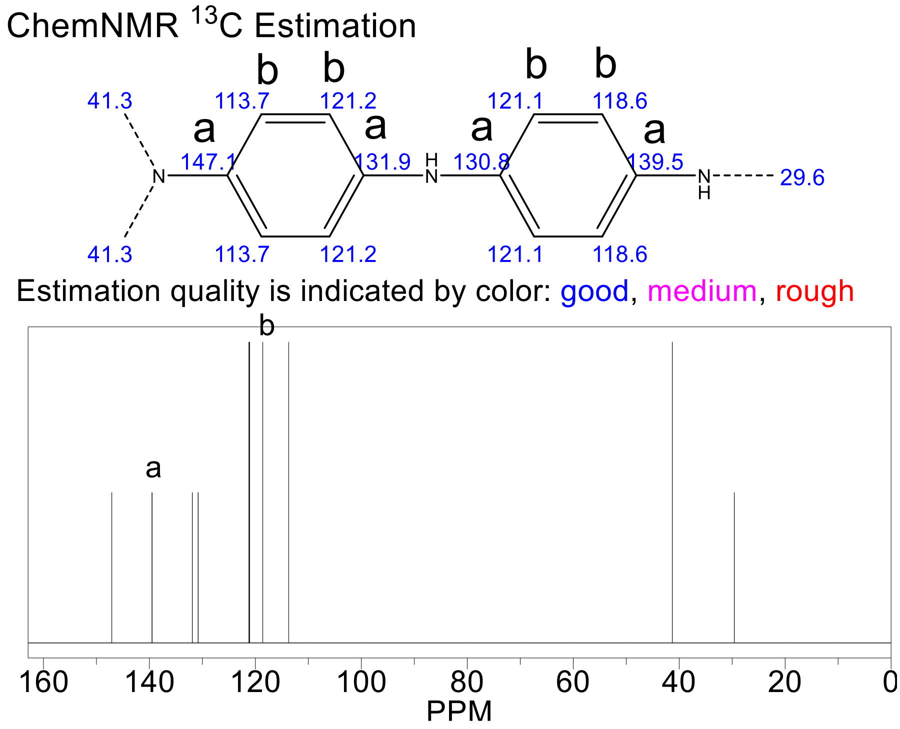


```
Protocol of the C-13 NMR Prediction: (Lib=S)

Node     Shift     Base + Inc.   Comment (ppm rel. to TMS)

C    131.9           128.5      1-benzene
                      14.7         1 -N-1:C*C*C*C*C*C*1
                     -10.5         1 -N(C)C
                      -0.8         general corrections
C    130.8           128.5      1-benzene
                      14.7         1 -N-1:C*C*C*C*C*C*1
                     -11.6         1 -N-C
                      -0.8         general corrections
C    147.1           128.5      1-benzene
                     -10.5         1 -N-1:C*C*C*C*C*C*1
                      16.0         1 -N(C)C
                      13.1         general corrections
C    139.5           128.5      1-benzene
                     -10.5         1 -N-1:C*C*C*C*C*C*1
                      15.0         1 -N-C
                       6.5         general corrections
CH   121.2           128.5      1-benzene
                     -10.6         1 -N-1:C*C*C*C*C*C*1
                       0.9         1 -N(C)C
                       2.4         general corrections
CH   121.1           128.5      1-benzene
                     -10.6         1 -N-1:C*C*C*C*C*C*1
                       0.8         1 -N-C
                       2.4         general corrections
CH   113.7           128.5      1-benzene
                       0.9         1 -N-1:C*C*C*C*C*C*1
                     -15.4         1 -N(C)C
                      -0.3         general corrections
CH   118.6           128.5      1-benzene
                       0.9         1 -N-1:C*C*C*C*C*C*1
                     -16.2         1 -N-C
                       5.4         general corrections
CH   121.2           128.5      1-benzene
                     -10.6         1 -N-1:C*C*C*C*C*C*1
                       0.9         1 -N(C)C
                       2.4         general corrections
CH   121.1           128.5      1-benzene
                     -10.6         1 -N-1:C*C*C*C*C*C*1
                       0.8         1 -N-C
                       2.4         general corrections
CH   113.7           128.5      1-benzene
                       0.9         1 -N-1:C*C*C*C*C*C*1
                     -15.4         1 -N(C)C
                      -0.3         general corrections
CH   118.6           128.5      1-benzene
                       0.9         1 -N-1:C*C*C*C*C*C*1
                     -16.2         1 -N-C
                       5.4         general corrections
CH3  41.3             -2.3      aliphatic
                      28.3         1 alpha -N
                       9.3         1 beta -1:C*C*C*C*C*C*1
                       9.4         1 beta -C
                      -3.4         general corrections
CH3  41.3             -2.3      aliphatic
                      28.3         1 alpha -N
                       9.3         1 beta -1:C*C*C*C*C*C*1
                       9.4         1 beta -C
                      -3.4         general corrections
CH3  29.6             -2.3      aliphatic
                      28.3         1 alpha -N
                       9.3         1 beta -1:C*C*C*C*C*C*1
                      -5.7         general corrections
```

Figure S5 Calculation results of the ChemNMR ^{13}C for the PTPA calculated by ChemDraw Professional 17.0.

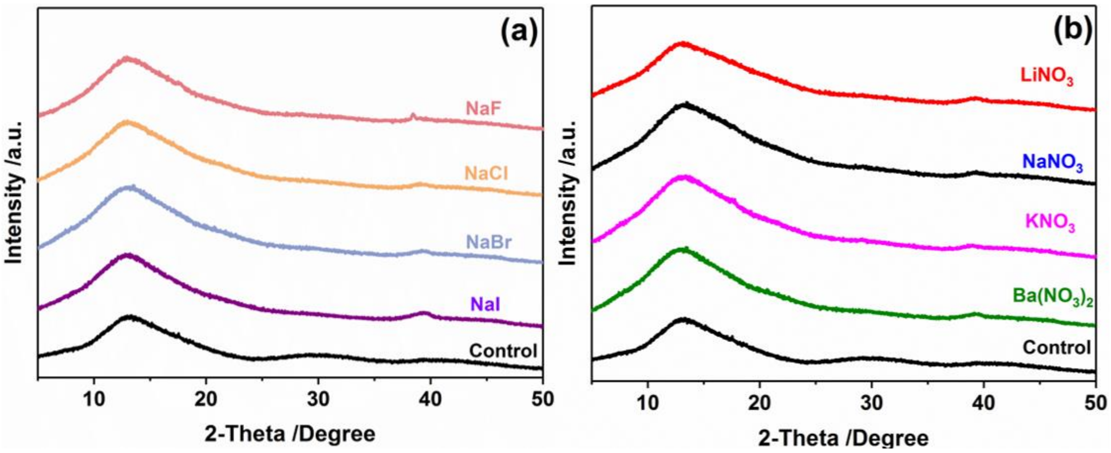

Figure S6 XRD diffractograms of PTPA networks after **BXJ** treatment with salts with varying (a) anions and (b) various cations (amorphous nature with main peak situated at around 13.0 °).

S4.1.5 N_2 adsorption and desorption isotherms, PSD and pore volume tuned using different dosages and solvents.

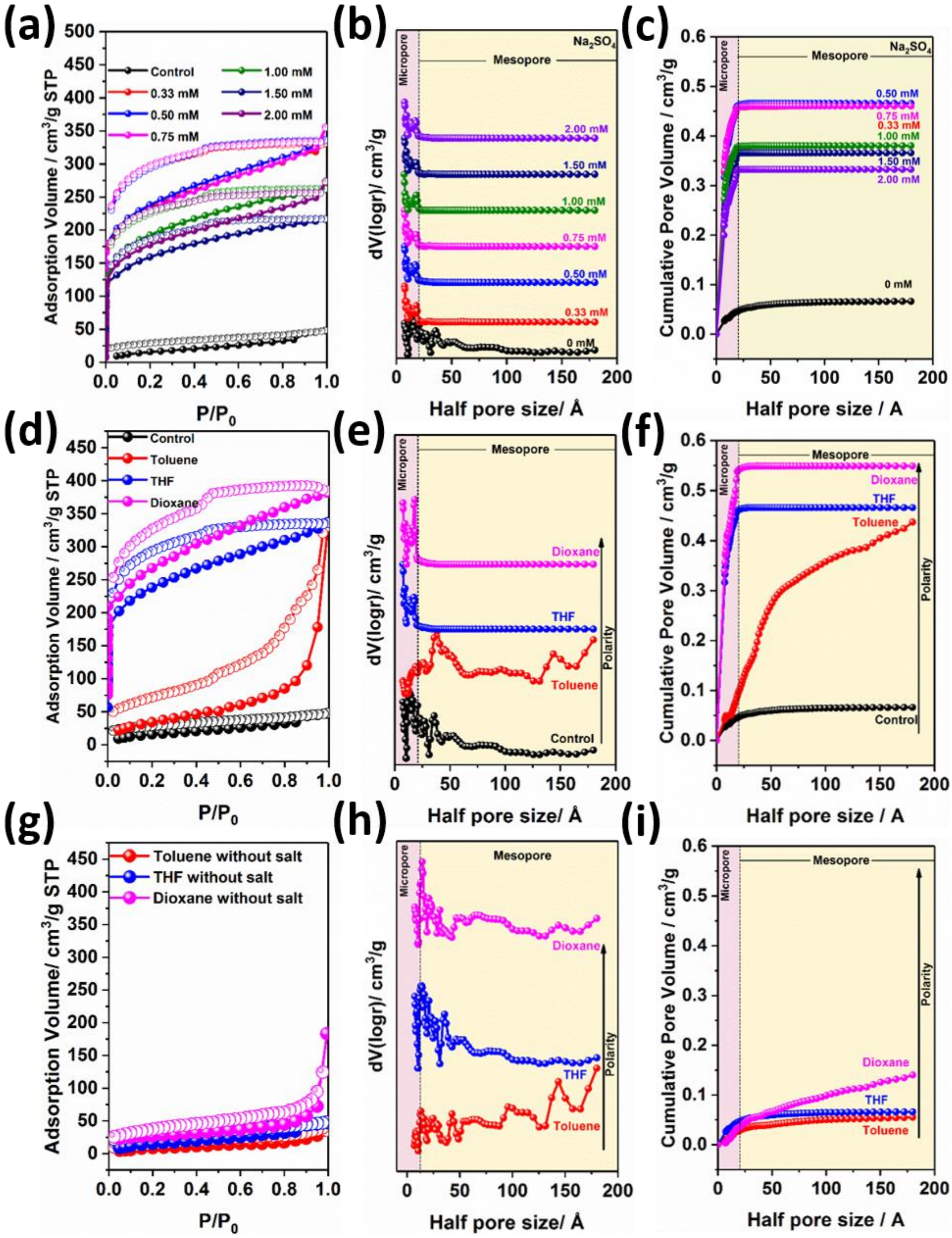

Figure S7 N_2 adsorption and desorption isotherms, nonlocal density functional theory-pore size distribution and cumulative pore volume of the PTPA networks tuned by salts with different ion dosage (a-c), solvents with 0.5 mM Na_2SO_4 (d-f) and solvents without salts (g-i) (the pink rectangular strips indicate the microporous region in the pore size distribution).

S4.1.6 Characterization for the mechanism of salt-tuning on the PTPA

1) FTIR

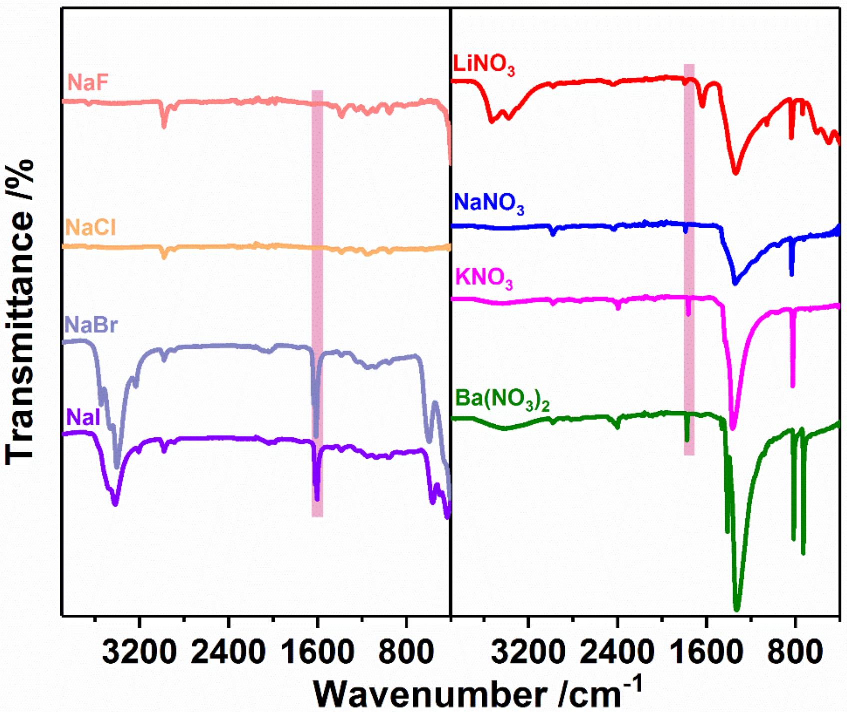

Figure S8 FTIR spectra of salts (the pink stripes indicate the characteristic peak ascribed to the salts which was also found in FTIR spectra of PTPA after salt tuning).

2) TEM

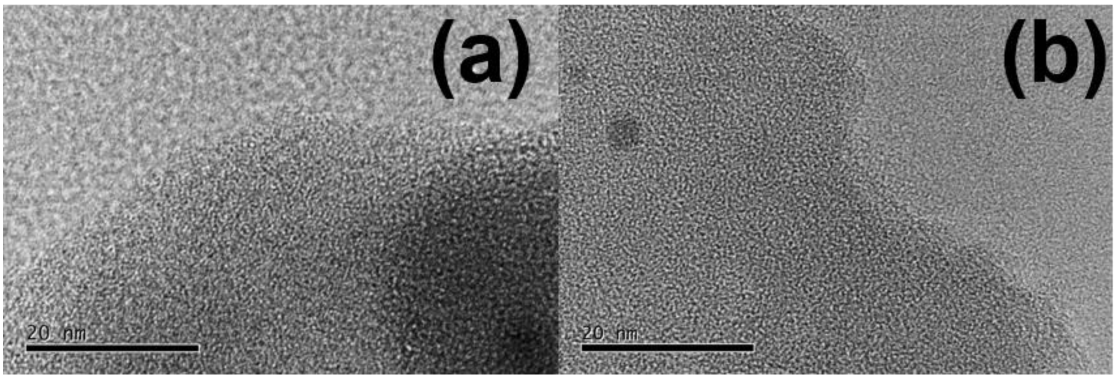

Figure S9 TEM images of PTPA before (a) and after **BXJ**-NaF tuning (b) (Scale bar 20 nm).

3) SEM

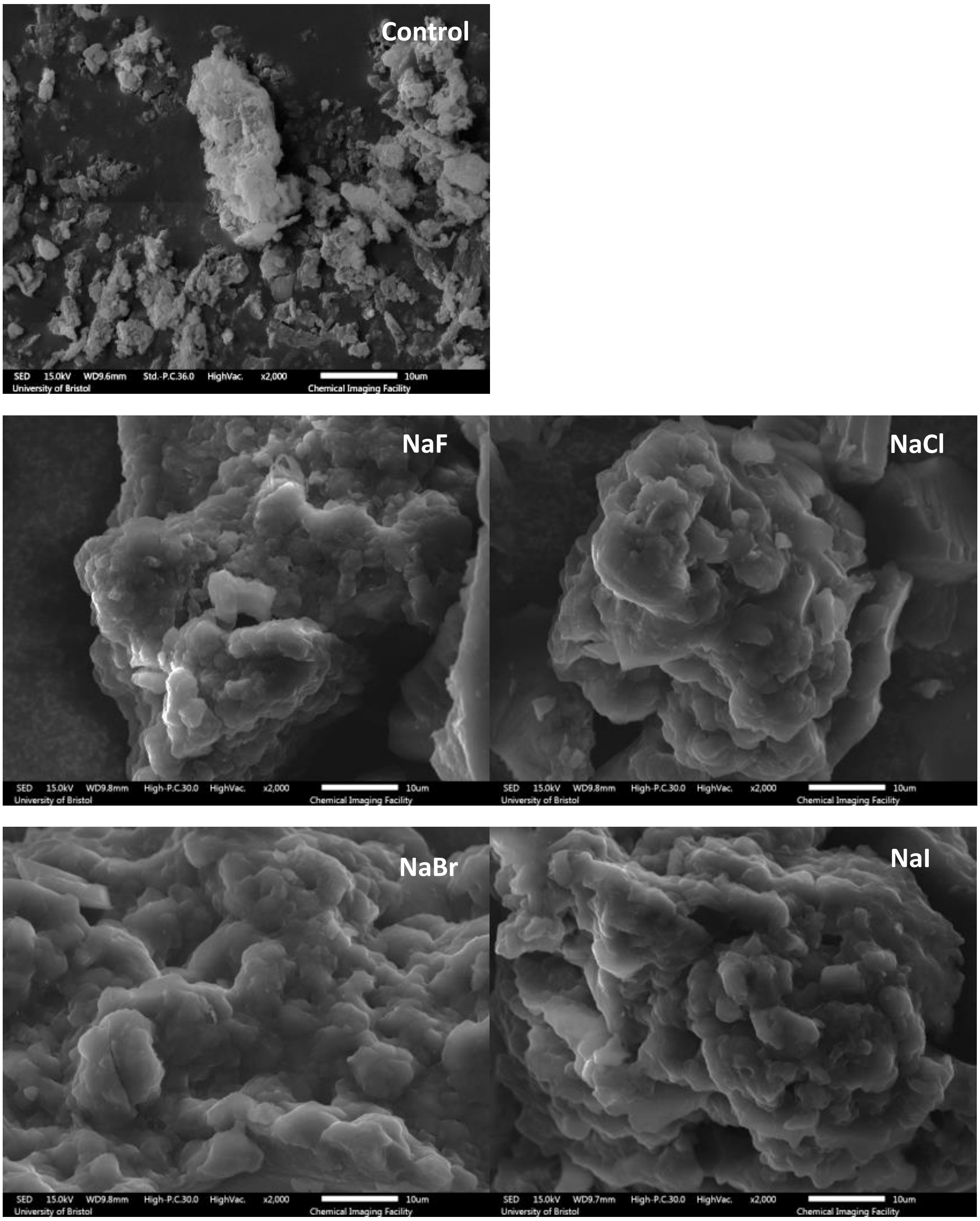

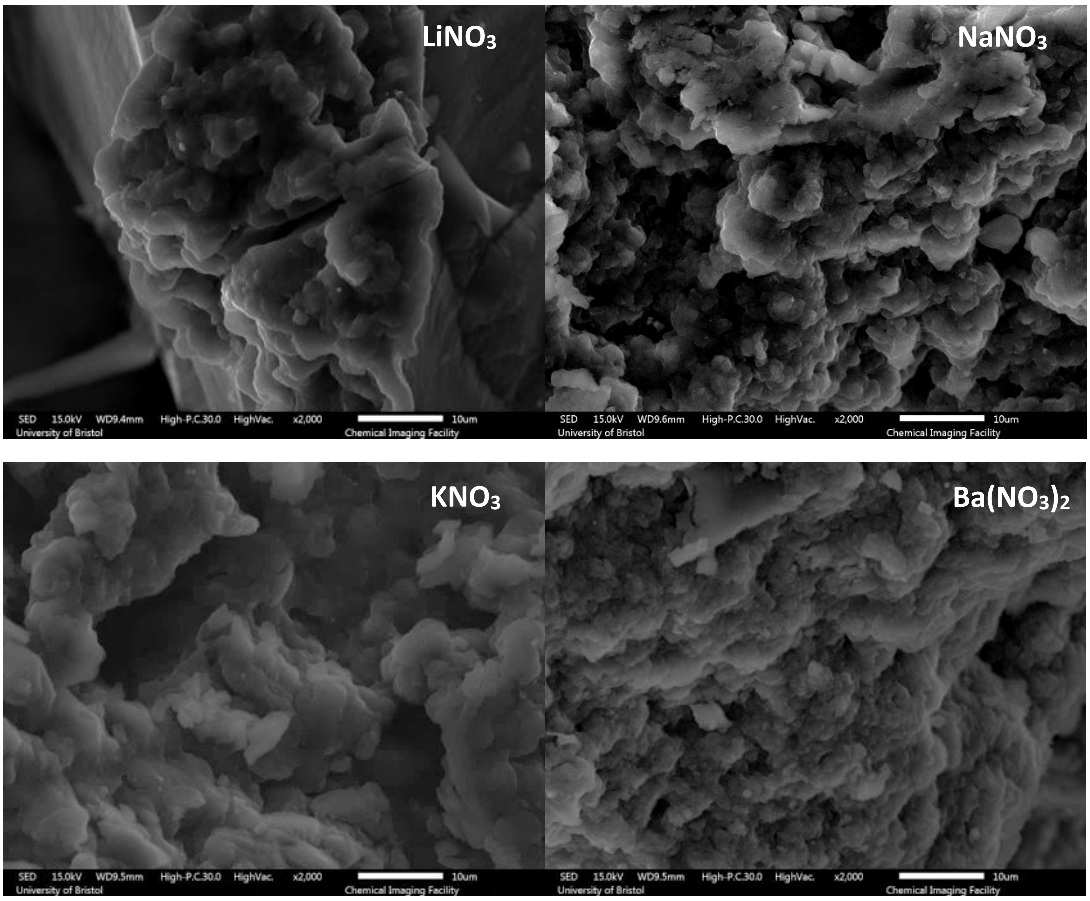

Figure S10 SEM images of the PTPA before and after salt tuning (Scale bar 10 μm).

4) UV-vis spectra

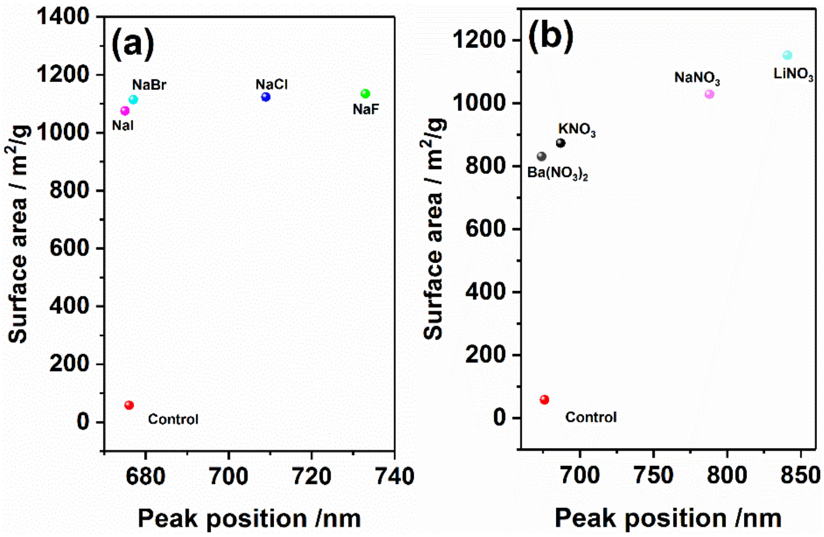

Figure S11 Correlation between UV-vis absorption peak and surface area of PTPA after salt tuning of different anions (a) and cations (b).

5) XPS

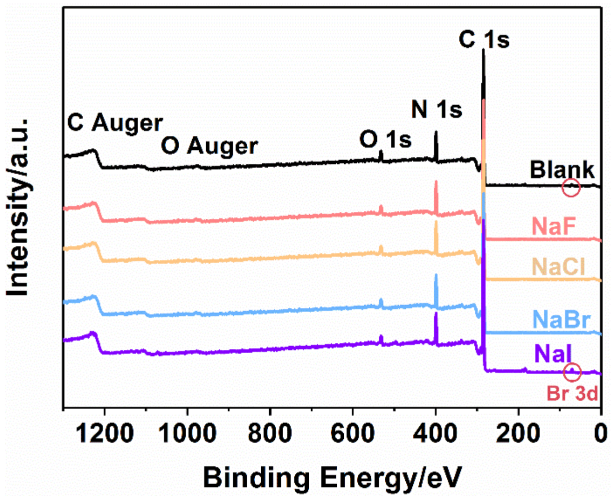

Figure S12 XPS spectra of PTPA before and after the tuning by BXJ-salt method.

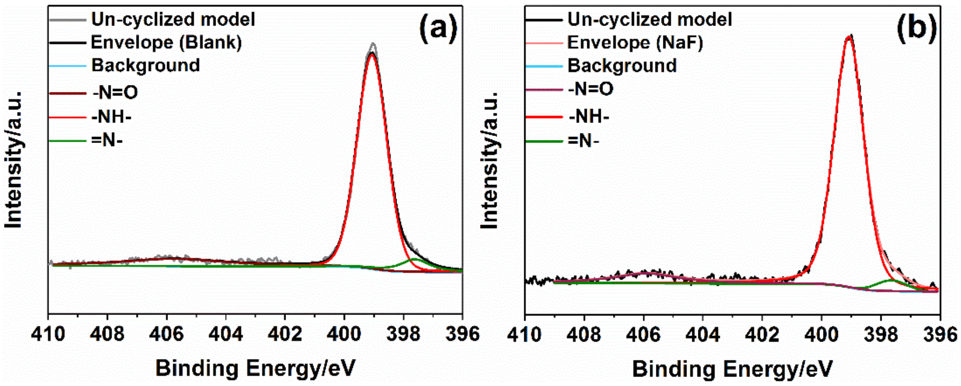

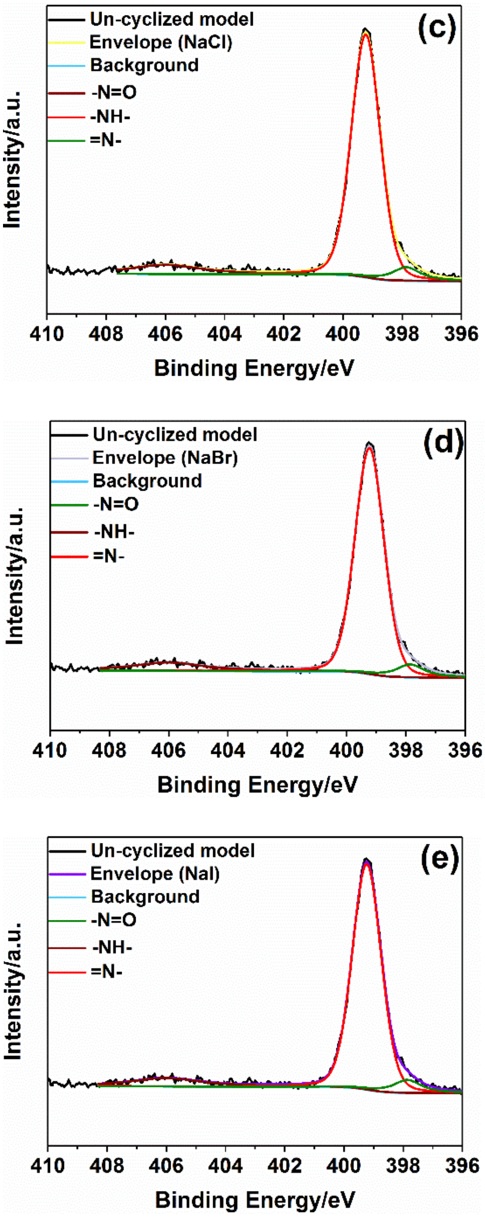

Salt	Peak position /eV	Peak assignment	Peak Area	Peak Area ratio	A(=N-)/ A(-NH-)
	406.1	-N=O	1035	8.20%	
**Blank**	399.1	-NH-	11214	88.3%	0.040
	397.6	=N-	449	3.53%	
	405.9	-N=O	1688	10.8%	
**NaF**	399.1	-NH-	12866	82.6%	0.080
	397.7	=N-	1028	6.60%	
	406.0	-N=O	1258	8.15%	
**NaCl**	399.2	-NH-	13277	86.0%	0.068
	397.7	=N-	896	5.85%	
	406.1	-N=O	1309	8.55%	
**NaBr**	399.1	-NH-	13122	85.7%	0.067
	397.8	=N-	879	5.75%	
	406.1	-N=O	1312	8.64%	
**NaI**	399.1	-NH-	12998	85.8%	0.065
	397.8	=N-	841	5.56%	

Figure S13 N 1s XPS spectra of PTPA before and after the tuning by **BXJ** method [11].

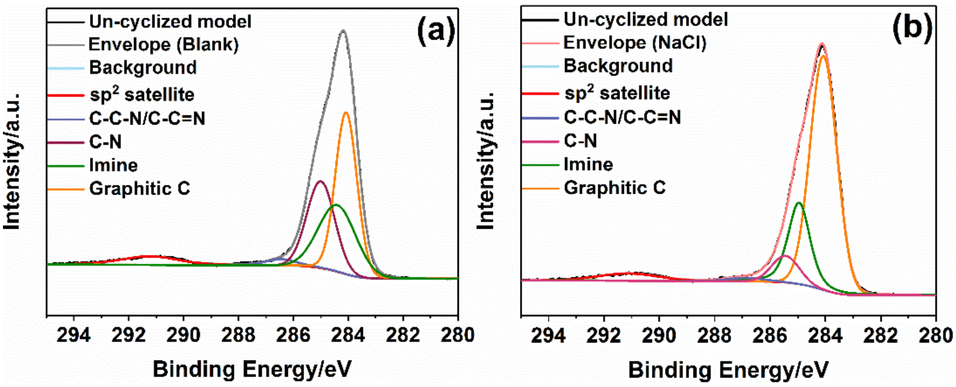

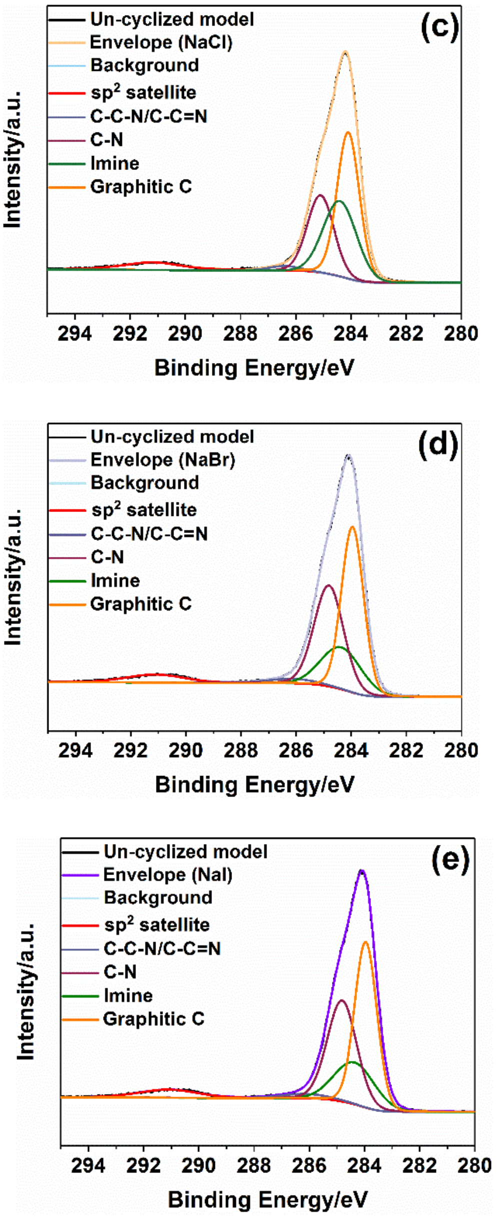

Salt	Peak position /eV	Peak assignment	Peak Area	Peak Area ratio
	291.3	sp^2 satellite	3681	5.99 %
	286.5	C-C=N	988	1.61 %
**Blank**	285.0	C-N	16215	26.4 %
	284.5	C-C	23805	38.7 %
	284.1	C-H	16778	27.3 %
	291.2	sp^2 satellite	2765	4.56%
	286.9	C-C=N	1787	2.95 %
**NaF**	285.5	C-N	4483	7.39 %
	284.9	C-C	12468	20.6 %
	284.1	C-H	39162	64.6 %
	291.3	sp^2 satellite	4262	4.89 %
	286.4	C-C=N	2208	2.51 %
**NaCl**	285.2	C-N	16809	19.3 %
	284.4	C-C	27449	31.5 %
	284.1	C-H	36362	41.8 %
	291.2	sp^2 satellite	3516	5.45 %
	286.2	C-C=N	1516	2.35 %
**NaBr**	284.9	C-N	11856	18.4 %
	284.5	C-C	21357	33.2 %
	284.0	C-H	26182	40.6 %
	291.3	sp^2 satellite	3015	5.23 %
	286.2	C-C=N	1010	1.77 %
**NaI**	284.9	C-N	10998	19.4 %
	284.4	C-C	20995	36.8 %
	284.0	C-H	21001	36.8 %

Figure S14 C 1s XPS spectra of PTPA before and after the tuning by **BXJ** method [6].

6) Calculation of HSPs of the PTPA

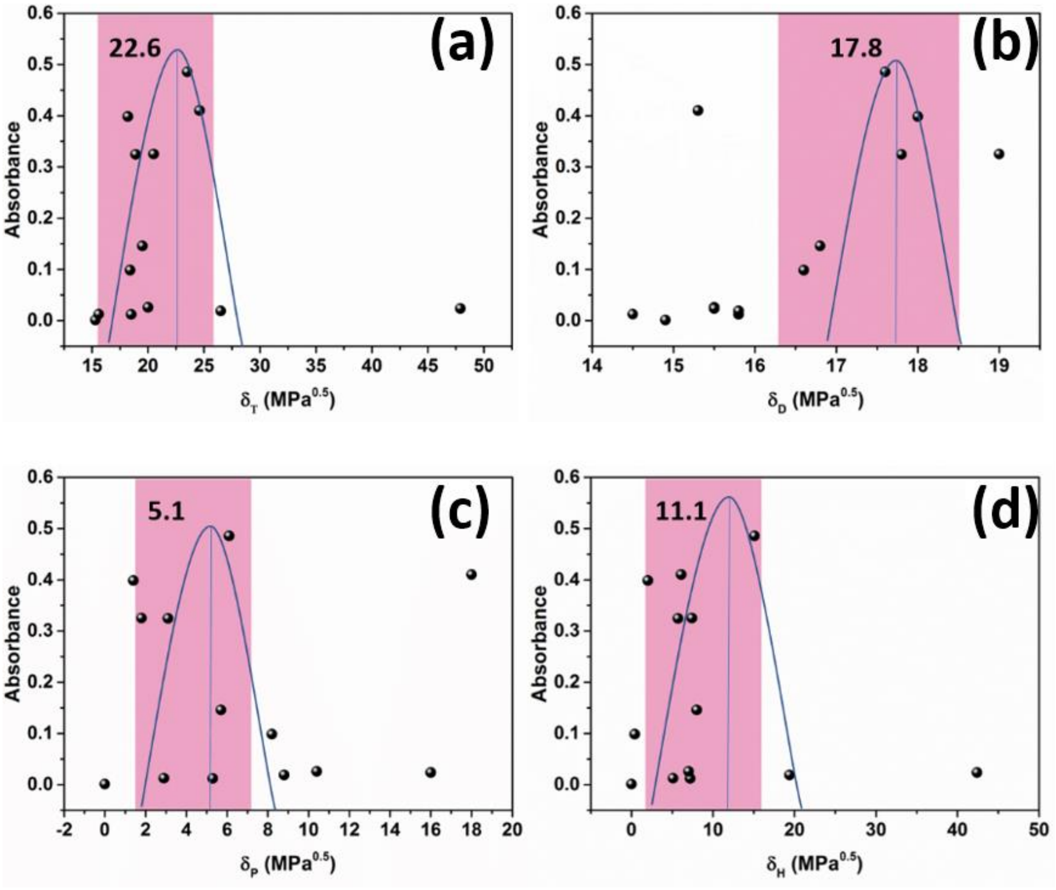

Figure S15 PTPA concentration measured with UV-vis plotted with Hansen solubility parameter of solvent: (a) total-, (b) disperse-, (c) polar- and (d) hydrogen bonding- solubility parameters (the pink strips were provided to highlight the maximum absorption region and the maximum adsorption peaks).[3b].

S4.1.8 CO_2 uptake of the PTPA

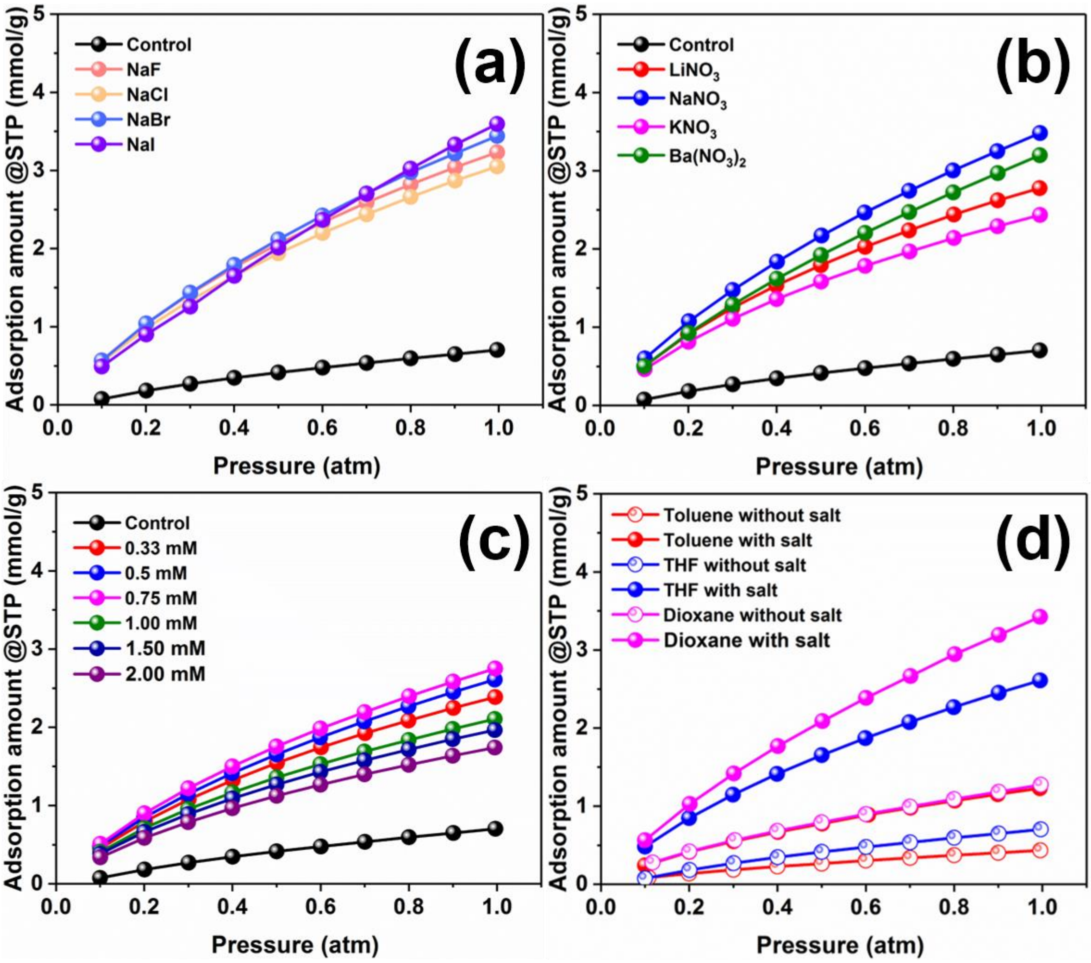

Figure S16 CO_2 uptake at 273 K of the PTPA tuned by salts with different anions (a) and cations (b) and with different ion dosage of Na_2SO_4 (c) in THF and in different solvents with/without 0.5 mM Na_2SO_4 (d).

S4.2 Supplementary tables

Table S1 Radii of the ions in the salt

	**Anion radius (Å)**		**Cation radius (Å)**
**F^-**	1.33	**Li^+**	0.76
**Cl^-**	1.84	**Na^+**	1.02
**Br^-**	1.95	**K^+**	1.33
**I^-**	2.20	**Ba^{2+}**	1.42

Table S2 BET surface area of some typical microporous polymers synthesized through different coupling reactions.

Coupling type	Samples	BET Surface area / m^2/g	Ref.
Sonogashira-Hagihara coupling	CMP-0	1018	[10]
	HCMP-1	842	[15]
	CMP-1	834	[10]
	CMP-2	634	[10]
	CMP-3	522	[10]
	CMP-5	512	[10]
	P2	510	[16]
Friedel–Crafts alkylation reactions	DBF-10	1800	[17]
	Vinylbenzyl chloride/divinylbenzene copolymers	1500	[18]
	DBF-25	1190	[17]
	Hypercrosslinked polystyrene	475-1000	[19]
	Hypercrosslinked polypyrroles	720	[20]
	Hypercrosslinked polyanilines	632	[21]
Suzuki coupling	Polyphenylene-based conjugated microporous polymer	1083	[22]
	SPOP-5	705	[23]
	SPOP-6	744	[23]
	Microporous bithiophene polymer	365	[24]
Yamamoto coupling	PAF-1	5660	[25]
	PPN-3	2840	[26]
	Poly-9,9’-spirobifluorene	1275	[27]
	P1	450	[16]
Fe^{3+}-catalysed oxidative polymerization	Microporous polycarbazole	2220	[28]
	Poly(tetraphenylmethane)	1980	[29]
	FCTCz	1845	[30]
	PTTPP	1522	[31]
	P(Fe-TTPP)	1248	[31]
	FCBCz	1067	[30]
	PS4AC2	1043	[32]
	PT4AC	762	[32]

Table S3 Porosity parameters and CO_2 uptake at 1 atm and 273 K of PTPA networks produced by the **BXJ** route with different anions, cations, ion dosage and solvents.

	**Ion radius (Å)**	**Surface area [a] (m^2/g)**	**Total Pore volume[b] (cm^3/g)**	**Micropore volume[c] (cm^3/g)**	**Ultramicropore volume[d] (cm^3/g)**	**CO_2 uptake at 273 K, 1 atm (mmol/g)**	**Ref**
Control	-	58	0.066	0.046	0.026	0.70	This work
NaF	1.33	1134	0.89	0.83	0.41	3.23	This work
NaCl	1.84	1123	0.67	0.65	0.33	3.05	This work
NaBr	1.95	1114	0.83	0.71	0.34	3.44	This work
NaI	2.20	1075	0.69	0.63	0.37	3.60	This work
$LiNO_3$	0.76	1152	0.62	0.61	0.34	2.78	This work
$NaNO_3$	1.02	1028	0.82	0.78	0.43	3.48	This work
KNO_3	1.33	873	0.53	0.52	0.33	2.43	This work
$Ba(NO_3)_2$	1.42	831	0.50	0.49	0.34	3.20	This work
0.33 mM Na_2SO_4	-	858	0.46	0.46	0.33	2.38	This work
0.50 mM Na_2SO_4	-	871	0.47	0.46	0.32	2.61	This work
0.75 mM Na_2SO_4	-	843	0.48	0.46	0.33	2.75	This work
1.00 mM Na_2SO_4	-	704	0.37	0.36	0.27	2.10	This work
1.50 mM Na_2SO_4	-	680	0.36	0.35	0.24	1.97	This work
2.00 mM Na_2SO_4	-	579	0.33	0.31	0.22	1.74	This work
Toluene without salts	-	38	0.055	0.028	0.009	0.43	This work
Toluene/0.5 mM Na_2SO_4	-	131	0.44	0.095	0.02	1.23	This work
THF without salts	-	58	0.066	0.046	0.026	0.70	This work
THF/0.5 mM Na_2SO_4	-	871	0.47	0.46	0.32	2.61	This work
Dioxane without salts	-	98	0.14	0.034	0.003	1.27	This work
Dioxane/0.5 mM Na_2SO_4	-	982	0.55	0.54	0.45	3.43	This work
CMP-1	-	834	0.53	0.33	N.A.	N.A.	[5]
COF-1	-	711	0.32	N.A.	N.A.	N.A.	[12]
CTF-1	-	791	0.40	N.A.	N.A.	N.A.	[13]
MOF-1	-	516	0.29	N.A.	N.A.	0.86	[14]

[a] Surface area calculated from the N_2 adsorption isotherm using the Brunauer-Emmett-Teller method. [b] The total pore volume calculated from the desorption branch of the N_2 isotherm using the NL-DFT method. [c] The micropore volume calculated from the desorption branch of the N_2 isotherm using the NL-DFT method for micropore (r<2 nm) volume. [d] The ultramicropore volume calculated from the desorption branch of the N_2 isotherm using the NL-DFT method for ultramicropore (r<0.7 nm) volume.

Table S4 Elemental analysis of the PTPA before and after salt tuning obtained from EDS.

	C		N		O		Br		Na/Li/K/Ba		F/Cl/I	
	**Atomic percentage %**	**σ[a]**	**Atomic percentage %**	**σ**	**Atomic percentage %**	**σ**	**Atomic percentage %**	**σ**	**Atomic percentage %**	**σ**	**Atomic percentage %**	**σ**
**PTPA**	87.65	0.62	8.05	0.66	2.33	0.17	0.42	0.09	1.55	0.07	N.A.	N.A.
**PTPA_NaF**	84.00	0.66	13.52	0.67	2.24	0.17	N.A.	N.A.	0.06	0.08	0.19	0.08
**PTPA_NaCl**	87.93	0.79	10.15	0.80	1.78	0.18	N.A.	N.A.	0.07	0.04	0.08	0.04
**PTPA_NaBr**	84.72	1.39	13.89	1.55	1.24	0.55	0.08	0.17	0.07	0.07	N.A.	N.A.
**PTPA_NaI**	84.25	1.82	15.38	2.07	0.95	0.20	N.A.	N.A.	N.A.	N.A.	N.A.	N.A.
**PTPA_$LiNO_3$**	84.95	1.07	12.19	1.09	2.86	0.28	N.A.	N.A.	N.A.	N.A.	N.A.	N.A.
**PTPA_$NaNO_3$**	78.34	0.67	16.98	0.70	4.49	0.21	N.A.	N.A.	0.19	0.03	N.A.	N.A.
**PTPA_KNO_3**	85.05	0.75	13.32	0.76	1.63	0.17	N.A.	N.A.	N.A.	N.A.	N.A.	N.A.
**PTPA_$Ba(NO_3)_2$**	88.94	0.58	4.04	0.62	4.16	0.20	N.A.	N.A.	2.87	0.42	N.A.	N.A.

[a] Sigma (**σ**) indicates the standard deviation for the results of each elements during the measurement.

Table S5 Summary of UV-vis adsorption peak position (PP) and surface area (SA)

Anion			Cation		
	**PP (nm)**	**SA (m^2/g)**		**PP (nm)**	**SA (m^2/g)**
**Control**	676	58	**Control**	676	58
**NaF**	733	1134	**$LiNO_3$**	841	1152
**NaCl**	709	1123	**$NaNO_3$**	788	1028
**NaBr**	687	1114	**KNO_3**	670	873
**NaI**	675	1075	**$Ba(NO_3)_2$**	668	831

Table S6 Hansen solubility parameters of 13 solvents used in this work; the PTPA dispersibility as defined by concentration after dispersion in 13 solvents and its Hansen solubility parameters.[2]

**Entry**	**Solvent/Polymer**	δ_T[a]	δ_D[b]	δ_P[c]	δ_H[d]	**Absorbance/a.u. (at 676 nm)**
1	Hexane	15.3	14.9	0	0	0.001252
2	Ethyl Acetate	18.5	15.8	5.3	7.2	0.012181
3	Diethyl ether	15.6	14.5	2.9	5.1	0.012563
4	Ethanol	26.5	15.8	8.8	19.4	0.018953
5	Toluene	18.2	18	1.4	2	0.398523
6	Dichloroethane	18.4	16.6	8.2	0.4	0.098581
7	Chloroform	18.9	17.8	3.1	5.7	0.324583
8	Acetone	20	15.5	10.4	7	0.025894
9	Isopropanol	23.5	17.6	6.1	15.1	0.485691
10	THF	19.5	16.8	5.7	8	0.145852
11	Acetonitrile	24.6	15.3	18	6.1	0.41021
12	Water	47.9	15.5	16	42.4	0.023696
13	1,4-Dioxane	20.5	19	1.8	7.4	0.325233
14	**PTPA**	22.6	17.8	5.1	11.1	

[a] δ_T suggests the total Hansen solubility parameter. [b] δ_D suggests the dispersion interaction parameter. [c] δ_P suggests the permanent dipole interaction parameter. [d] δ_H suggests the hydrogen-bonding interaction parameter.

Reference

[1] Y. Liao, J. Weber, C. F. J. Faul, *Chem. Commun.* **2014**, *50*, 8002-8005.

[2] D. J. Hansen C., Kontogeorgis G., Panayiotou C., Willliams L., Poulsen T., Priebe H., Redelius P. , *Boca Raton: CRC Press* **2007**.

[3] a) H. Launay, C. M. Hansen, K. Almdal, *Carbon* **2007**, *45*, 2859-2865; b) S. Ata, T. Mizuno, A. Nishizawa, C. Subramaniam, D. N. Futaba, K. Hata, *Scientific Reports* **2014**, *4*, 7232.

[4] J. Chen, N. Wang, Y. Liu, J. Zhu, J. Feng, W. Yan, *Synth. Met.* **2018**, *245*, 32-41.

[5] J.-X. Jiang, F. Su, A. Trewin, C. D. Wood, N. L. Campbell, H. Niu, C. Dickinson, A. Y. Ganin, M. J. Rosseinsky, Y. Z. Khimyak, A. I. Cooper, *Angew. Chem.* **2007**, *119*, 8728-8732.

[6] S. N. Kumar, F. Gaillard, G. Bouyssoux, A. Sartre, *Synth. Met.* **1990**, *36*, 111-127.

[7] F. S. Macintyre, D. C. Sherrington, *Macromolecules* **2004**, *37*, 7628-7636.

[8] a) A. I. Cooper, A. B. Holmes, *Adv. Mater.* **1999**, *11*, 1270-1274; b) A. K. Hebb, K. Senoo, R. Bhat, A. I. Cooper, *Chem. Mater.* **2003**, *15*, 2061-2069; c) C. D. Wood, A. I. Cooper, *Macromolecules* **2001**, *34*, 5-8.

[9] W. Kangwansupamonkon, S. Damronglerd, S. Kiatkamjornwong, *J. Appl. Polym. Sci.* **2002**, *85*, 654-669.

[10] J.-X. Jiang, F. Su, A. Trewin, C. D. Wood, H. Niu, J. T. A. Jones, Y. Z. Khimyak, A. I. Cooper, *J. Am. Chem. Soc.* **2008**, *130*, 7710-7720.

[11] E. T. Kang, K. G. Neoh, K. L. Tan, *Prog. Polym. Sci.* **1998**, *23*, 277-324.

[12] A. P. Côté, A. I. Benin, N. W. Ockwig, M. O'Keeffe, A. J. Matzger, O. M. Yaghi, *Science* **2005**, *310*, 1166-1170.

[13] P. Kuhn, M. Antonietti, A. Thomas, *Angew. Chem. Int. Ed.* **2008**, *47*, 3450-3453.

[14] Z. Guo, H. Xu, S. Su, J. Cai, S. Dang, S. Xiang, G. Qian, H. Zhang, M. O’Keeffe, B. Chen, *Chem. Commun.* **2011**, *47*, 5551-5553.

[15] J.-X. Jiang, F. Su, H. Niu, C. D. Wood, N. L. Campbell, Y. Z. Khimyak, A. I. Cooper, *Chem. Commun.* **2008**, 486-488.

[16] J. Weber, A. Thomas, *J. Am. Chem. Soc.* **2008**, *130*, 6334-6335.

[17] M. G. Schwab, A. Lennert, J. Pahnke, G. Jonschker, M. Koch, I. Senkovska, M. Rehahn, S. Kaskel, *J. Mater. Chem.* **2011**, *21*, 2131-2135.

[18] B. Li, X. Huang, L. Liang, B. Tan, *J. Mater. Chem.* **2010**, *20*, 7444-7450.

[19] M. P. Tsyurupa, V. A. Davankov, *React. Funct. Polym.* **2006**, *66*, 768-779.

[20] J. Germain, J. M. J. Fréchet, F. Svec, *Chem. Commun.* **2009**, 1526-1528.

[21] J. Germain, J. M. J. Fréchet, F. Svec, *J. Mater. Chem.* **2007**, *17*, 4989-4997.

[22] L. Chen, Y. Honsho, S. Seki, D. Jiang, *J. Am. Chem. Soc.* **2010**, *132*, 6742-6748.

[23] Q. Chen, Q. Wang, M. Luo, L.-J. Mao, C.-G. Yan, Z.-H. Li, B.-H. Han, *Polymer* **2012**, *53*, 2032-2037.

[24] D. Xiao, Y. Li, L. Liu, B. Wen, Z. Gu, C. Zhang, Y. S. Zhao, *Chem. Commun.* **2012**, *48*, 9519-9521.

[25] T. Ben, H. Ren, S. Ma, D. Cao, J. Lan, X. Jing, W. Wang, J. Xu, F. Deng, J. M. Simmons, S. Qiu, G. Zhu, *Angew. Chem. Int. Ed. Engl.* **2009**, *48*, 9457-9460.

[26] W. Lu, D. Yuan, D. Zhao, C. I. Schilling, O. Plietzsch, T. Muller, S. Bräse, J. Guenther, J. Blümel, R. Krishna, Z. Li, H.-C. Zhou, *Chem. Mater.* **2010**, *22*, 5964-5972.

[27] J. Schmidt, M. Werner, A. Thomas, *Macromolecules* **2009**, *42*, 4426-4429.

[28] Q. Chen, M. Luo, P. Hammershøj, D. Zhou, Y. Han, B. W. Laursen, C.-G. Yan, B.-H. Han, *J. Am. Chem. Soc.* **2012**, *134*, 6084-6087.

[29] S. Yao, X. Yang, M. Yu, Y. Zhang, J.-X. Jiang, *Journal of Materials Chemistry A* **2014**, *2*, 8054-8059.

[30] X. Yang, M. Yu, Y. Zhao, C. Zhang, X. Wang, J.-X. Jiang, *RSC Advances* **2014**, *4*, 61051-61055.

[31] J. Xia, S. Yuan, Z. Wang, S. Kirklin, B. Dorney, D.-J. Liu, L. Yu, *Macromolecules* **2010**, *43*, 3325-3330.

[32] S. Yuan, S. Kirklin, B. Dorney, D.-J. Liu, L. Yu, *Macromolecules* **2009**, *42*, 1554-1559.
